# Pupil size in the evaluation of static and dynamic stimuli in peripheral vision

**DOI:** 10.1371/journal.pone.0250027

**Published:** 2021-05-10

**Authors:** Stefanie Klatt, Benjamin Noël, Andreas Brocher

**Affiliations:** 1 Institute of Sports Science, University of Rostock, Rostock, Germany; 2 Institute of Exercise Training and Sport Informatics, German Sport University Cologne, Cologne, Germany; 3 CRC Prominence in Language, University of Cologne, Cologne, Germany; 4 Institut für Deutsche Sprache und Literatur I, Cologne, Germany; Groningen University, NETHERLANDS

## Abstract

It has been evidenced that in attention-window tasks, the participants fixate on the center of a screen while inspecting two stimuli that appear at the same time in parafoveal vision. Such tasks have successfully been used to estimate a person’s breadth of attention under various conditions. While behavioral investigations of visual attention have often made use of response accuracy, recent research has shown that the pupil size can also be used to track shifts of attention to the periphery. The main finding of previous studies is that the harder the evaluation of the stimuli becomes, e.g., because they appear farther away from the central fixation point, the stronger the pupils dilate. In this paper, we present experimental data suggesting that in an attention-window task, the pupil size can also be used to assess whether the participants attend to static, non-moving, or dynamic, moving stimuli. That is, regression models containing information on presentation mode (static vs. dynamic) and the visual angle between spatially separated stimuli better predict accuracy of perception and pupil dilation than model without these sources of information. This finding is useful for researchers who aim at understanding the human attentional system, including potential differences in its sensitivity to static and dynamic objects.

## Introduction

It is widely known that the size of the pupil changes as a function of brightness (e.g., [1]; for a review see [2]), success in memory retrieval (e.g., [3]), and effort (e.g., [4]). Mathôt et al. [5] even used the link between pupil light response and covert attention to letters of alternating brightness, whereas Binda and Gamlin [6] suggested a connection of attentional processes and pupil light responses.

Recently, Brocher et al. [7] used pupil size to investigate covert shifts of attention. However, unlike Mathôt et al. [5], who showed that pupil-light response is associated with covert attention, Brocher et al. [7] explored the well-established link between pupil dilation, on the one hand, and task effort on the other without making use of foveal vision to two spatially separated stimuli. They used a modified version of the attention-window task [8, 9] in the form of an attention-demanding conjunction task which requires attentive processing of two stimuli presented in the peripheral vision. Brocher et al. [7] found that pupil size correlates with the size of the visual angle at which the two stimuli are presented relative to the fixation point: The further away from the fixation point the two to-be-attended-to objects appeared, the stronger the pupils dilated. Furthermore, reacting to low contrast stimuli led to larger pupils than reacting to high contrast stimuli, and the inspection of such stimuli in the peripheral vision resulted in overall larger pupils than the mere detection of these stimuli. These findings complement previous studies showing that pupil sizes are modulated by attentional load when using attention-demanding visuo-spatial tasks [10].

In visual search tasks (e.g., [11]) as well as in visuomotor tasks (e.g., [12]), researchers have demonstrated that pupil size can provide a general metric to assess attentional load. With regard to their own results, Brocher and colleagues [7] also concluded that the attention-window task, in combination with measurement of the pupil size, is a promising tool for testing specific features of the various processes that underlie perception and attentional shifts to peripheral vision. The great advantage of this reported paradigm is that the assessment of a participant’s breadth of attention does not hinge on the output of a specific task, such as the accuracy with which objects in peripheral vision are evaluated. The paradigm could therefore, in principle, involve a variety of tasks, and the participants would not necessarily need to be directed to attend to one or the other stimulus in the periphery (i.e., as long as task demands do not afford different amounts of effort). Indeed, it seems that the only precondition for a successful use of the paradigm is that the attentional shifts of interest correlate with differences in effort: More effort is required for the evaluation of stimuli that appear further away from eye fixation compared to those closer to the eye fixation (for a review, see [13]).

In this paper, we test a potential extension of the paradigm reported by Brocher et al. [7]. We investigate if it can be used to track whether a person attends to static, non-moving objects, or a dynamic, moving objects. As the visual world contains both static and dynamic components, the human visual system is required to respond to a wide variety of stimuli. The ability to identify moving objects (particularly in the horizontal dimension of the attentional focus) is considered essential for many daily activities, such as participating in sports activities and driving a car (e.g., [14, 15]).

Work by Ludvigh and Miller [16] has already described the ability of the eyes to resolve stimuli that move relative to an observer as “dynamic visual acuity”. It is interesting to note that most methods that have been used to investigate specific perceptual and attentional capabilities or processes of the cognitive system, have focused on interactions with static stimuli; and that the various studies that have compared static and dynamic stimuli suggest that dynamic events show an overall higher sensitivity over the visual field than static events (see e.g., [17]). However, it has also been claimed that attending to dynamic stimuli comes at a higher cost than static stimuli [18].

Specific differences between attentional shifts to static vs. dynamic objects have not yet been explored in detail, and it is important to point out that the purpose of the present study was not to shed light on the processes that might underlie these differences. Rather, our goal was to test an online experimental paradigm that could help researchers focus on specific properties of the attentional system, particularly with respect to the difference between static and dynamic visual acuity. As previous research has found that dynamic stimuli determine stronger brightness effects compared to static ones (e.g., [19]), we predict relatively large changes in the pupil size when dynamic stimuli are presented than the corresponding static ones.

## Method

### Participants

Twenty subjects (14 females) between the ages of 18 and 35 years participated in the study for course credit. All participants had normal or corrected-to-normal vision. All participants gave their written informed consent prior to their inclusion in the study. The study has been approved by the local ethical committee (ethics commission of the German Sport University Cologne) and has, therefore, been performed in accordance with the ethical standards laid down in the 1964 Declaration of Helsinki and its later amendments.

### Materials

We used the same 150 target stimuli that were used by Brocher et al. [7] in their Experiment 1. These stimuli were squares, 64 x 64 pixels in size, containing black or white triangles or circles that were 30 x 30 pixels in size each. Importantly, the stimuli varied with respect to both the number of white triangles that they contained and the way that the triangles and circles were arranged within the squares. A stimulus was composed of four objects, which could be filled circles or triangles that were black or white. Each object could be any combination of the forms (circle, triangle) and shading (white, black), selected at random on each of the 150 fixed configurations that were presented to every participant exactly once (RGB for white objects: 254, 254, 254; RGB for black objects: 0, 0, 0). In every trial, each stimulus had an equal probability (20%) of including zero, one, two, three, or four white triangles.

Two target stimuli appeared equidistant to the central fixation point in each trial, and the participants’ task was to report how many white triangles they saw within each of these two stimuli. By requiring the participants to detect, not just the shape or the shading of the elements, but rather, the conjunction of both (i.e., identifying the white triangles), the attention-window task is, in its nature, an attention-demanding task [20]). The target stimuli were always preceded by pre-cues which were black circles extending over 30 pixels. They always appeared at the locations where the target stimuli would appear immediately afterwards, and importantly, pre-cues and target stimuli always appeared horizontally to the fixation point.

The participants were seated 60cm away from a computer screen and instructed to fixate on the center of the screen (cf. [21]); the target stimuli could appear at five different angles relative to the central fixation point (30 trials per angle): 12.5°, 20°, 27.5°, 35°, and 42.5°. There were two presentation modes: In the static mode, stimuli appeared and disappeared at the very same locations (no movement); in the dynamic mode, pre-cues and target stimuli appeared 7.5° closer to the central fixation and immediately moved outwards to the endpoints creating the respective presentation angles (12.5°, 20°, 27.5°, 35°, or 42.5°). For example, when the pre-cues and subsequent target stimuli appeared at 5° to the fixation point, they would move towards the 12.5° angle and then disappear. This trial would then count as a 12.5° angle trial. The participants worked on 75 trials each, in the static and the dynamic mode, with 15 trials of each angle in each mode. Trials of both modes (static, dynamic) were alternated and presented in random order in one session.

### Procedure

**Fig 1:**
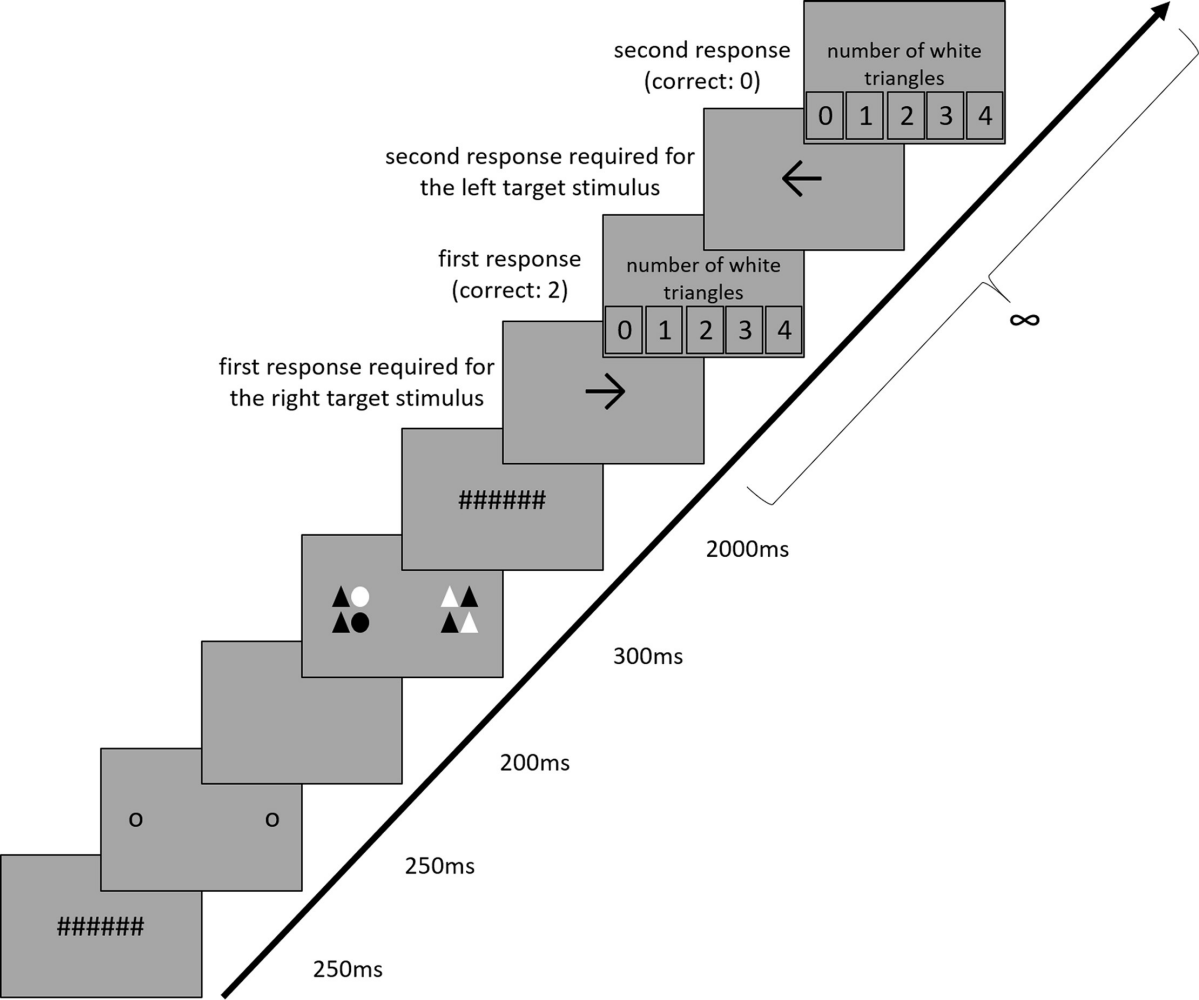
Example of a trial in the experiment. The size ratio of the stimuli has been adjusted to better illustrate the procedure.

The participants received written information that the study was about peripheral vision and the question of how well objects can be perceived in the periphery. Afterwards calibration of the eye tracker took place. In each trial, the participants were asked to fixate on the center of the screen and inspect the various objects only peripherally, with no fixation on these objects. Each trial started with a screen indicating to the participants which mode was about to follow. In the static mode, the words *No Movement* appeared on the display. In the dynamic mode, the word *Movement* appeared. All the displays in the experiment were dark grey, with RGB settings of 153, 153, 153, and the illuminance, measured at the participants’ right eye, was maintained at an average of 26 lx. An example of a trial is provided in Fig 1.

After the participants pressed the button on the mouse, seven hash marks appeared at the center of the screen. After 250ms, the two pre-cues appeared, either at one of the five angles (static mode) or 7.5° closer to the fixation point than the respective angle (dynamic mode). The pre-cues lasted for 250ms and were then replaced by a blank screen. After 200ms, the two target stimuli appeared and, in the static mode, remained at their location for 300ms or, in the dynamic mode, moved towards their landing positions within 300ms. Next, the seven hash marks re-appeared and stayed on screen for 2000ms. During these 2000ms, we measured the size of the right pupil in response to the target stimuli inspection.

After the final fixation screen, a black arrow emerged at the center of the screen. When it pointed to the left, the participants were asked to provide their response for the target stimulus that appeared at the left of the center; when it pointed to the right, the participants were asked to provide their response for the target stimulus that appeared at the right of the center. Next, five numbers appeared on the screen and the participants clicked on the number that represented their response. For example, when the participants counted three white triangles in the left target stimulus and the first arrow pointed to the left, participants were supposed to click on the number three. After the first response, another arrow appeared which pointed in the opposite direction, so that participants could provide their response for the second stimulus. The order of appearance of the left and right arrows was randomized between trials. After the second response and a delay of 2000ms the subsequent trial started. The experiment started with 15 random practice trials.

### Recording and analysis

The experiment was conducted using an EyeLink 1000, configured with an Intel Core i7-4770, 3.4 GHz, 4 GB RAM, running Windows 7 SP1, and a ViewSonic VS 12538 monitor (screen resolution during testing was 1,024 × 768). Vision was binocular but we only recorded the pupil size of the right eye, at a rate of 250 Hz. For the pupil size measure, we calculated the maximum size of the pupil for a baseline and a target window. As the baseline measure, we determined the peak pupil size for the 250ms that the pre-cues were presented. As the measure of stimuli evaluation, we calculated the peak pupil size for the 2000ms after the target stimuli presentation. We extracted the maximum pupil size of the trial baseline and target window for each participant individually and then averaged that value with the size values 50ms before and 50ms after the respective maximum (cf. [3, 7]). Finally, we subtracted the baseline measure from the target measure as the baseline-corrected peak pupil size.

Before the statistical analyses, we excluded all data points that resulted from blinks and those which deviated three standard deviations or more from the trial’s mean. This resulted in a total loss of 3.3% of the data. A visual inspection of the data confirmed that the participants had kept their eye fixated on the screen center. Furthermore, the removal of the fixation data exceeding three standard deviations of a trial’s mean also removed all the data where the participants looked at the target stimuli directly.

In addition to the pupil size data, we also calculated the accuracy with which the participants evaluated the two target stimuli. We counted any response for which both target stimuli were evaluated correctly as correct, and all other responses as incorrect. We included all responses, correct and incorrect, in the analysis of the pupil dilation.

For the pupil size data and the response data, we fitted several linear and generalized mixed models, respectively, successively increasing model complexity. For the pupil size data, the dependent measure was the baseline-corrected peak pupil size, for the accuracy data the dependent measure was the proportion of correct vs. incorrect responses. The first model in the analyses of either measure only included an intercept; the second model included the factor presentation mode (static or dynamic); the third model the linear combination of presentation mode and angle; and the fourth model the interaction of presentation mode and angle as predictors. Both, angle and presentation mode were sum-coded prior to model fitting. In addition, all models included random intercepts for the participants and the target stimuli, as well as random slopes for angle, presentation mode, and their interaction as by-participant and by-stimuli random slopes. When adding by-stimulus random effects, we considered a stimulus for each unique combination of shapes and their spatial arrangements. There were 150 such stimuli, sub-sampled from the larger number of possible combinations, and each of these was presented exactly once to each participant. Consequently, the model had 150 by-stimuli random intercepts and random slopes. The random slopes were included to acknowledge that not only the group mean of the included dependent variables are likely to vary, but also the results are likely to differ across different participants and target stimuli; for example, different effect sizes might occur for the different stimuli of the experiment.

To test for statistical reliability of the factors’ presentation mode and angle in both the analysis of pupil dilation and the analysis of response accuracy, we first compared the intercept-only model to the presentation mode model, then the presentation mode model to the presentation mode + angle model and, finally, the presentation mode + angle model to the interaction model, using log-likelihood ratio tests. Generally speaking, if a specific factor, or the combination or interaction of two factors, significantly contributes to the variance observed in the data, we expect model fit to significantly improve with inclusion of that factor (or with inclusion of the combination or the interaction of the two factors). For example, if the mode of stimuli presentation differently affects the size of the pupil, we should find the presentation mode model to be a better fit to the data, i.e., to explain more variance in the data, than the intercept-only model.

## Results

**Fig 2:**
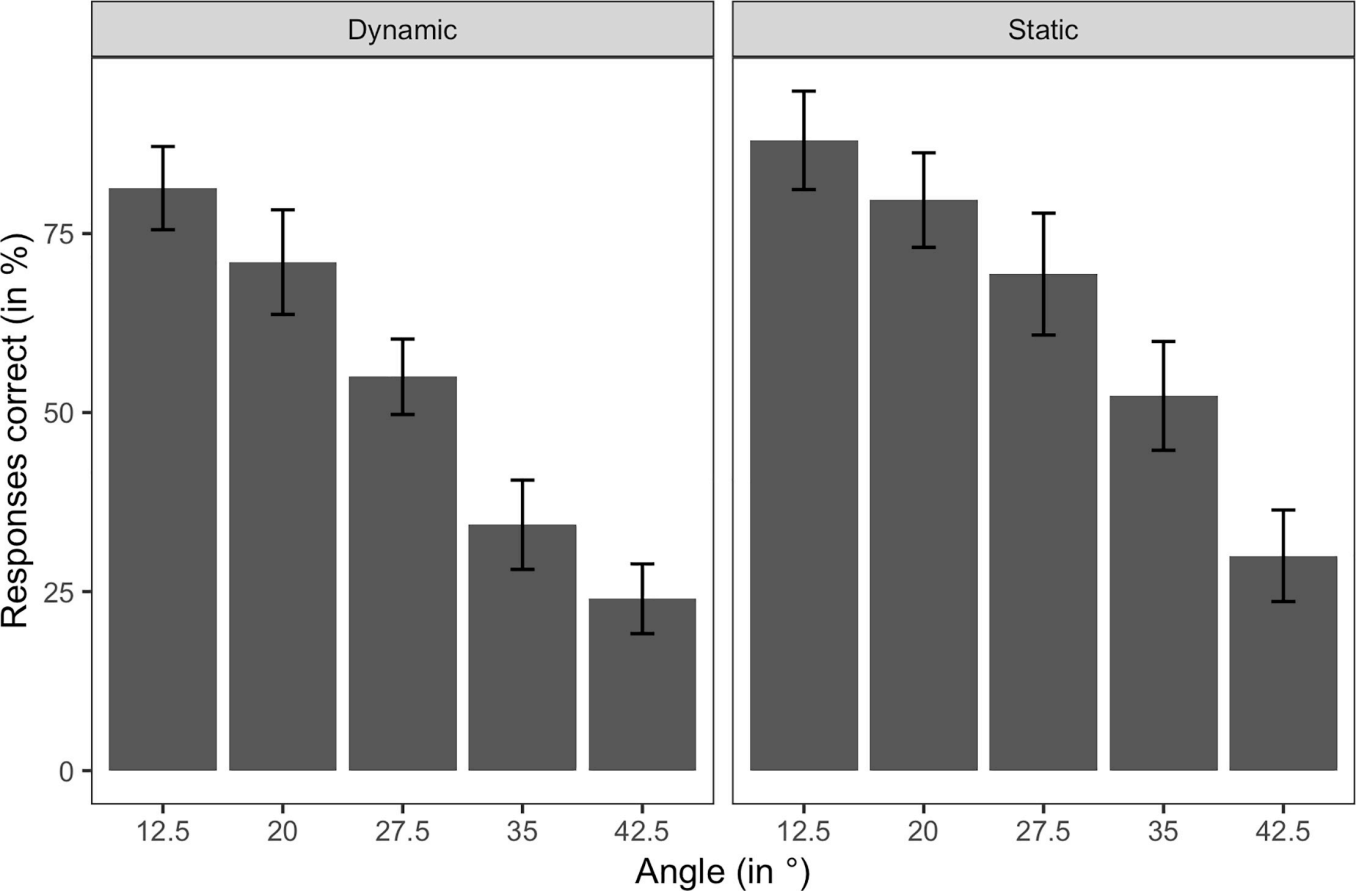
Mean response accuracy per angle. Bars indicate the proportion of correct responses. Error bars provide 95% confidence intervals.

We will first discuss the response data results and then turn to the pupil size data results. As can be seen in Fig 2, participants’ accuracy decreased with increasing angle; this is true for both the static and the dynamic mode. However, for all the five angles, the accuracy was slightly higher for the static than the dynamic mode. This is also reflected by results of a 2 (presentation mode: static, dynamic) x 5 (angle: 12.5°, 20°, 27.5°, 35°, and 42.5°) repeated measures ANOVA on the accuracy data which showed a significant main effects of presentation mode, *F*(1, 19) = 6.346, *p* = .021, and angle, *F*(4, 76) = 5.928, *p* < .001.

Model comparison revealed that the presentation mode model (*AIC* = 3291.7) fit the data better than the intercept-only model, Χ^2^(1) = 60.88, *p* < .001, (*AIC* = 3350.6). When we also included angle as the dependent variable, model fit improved further, Χ^2^(4) = 118.95, *p* < .001, *AIC* = 3180.8 (*R*^*2*^ = 0.151, *b*_*0*_ = 0.418, *b*_*presentation mode*_ = 0.545, *b*_*angle*_ = 2.23, *ps* < .001). When we also included the Presentation mode x Angle interaction in the model, no further improvement occurred, Χ^2^(4) = 4.88, *p* = .30, (*AIC* = 3183.9).

**Fig 3:**
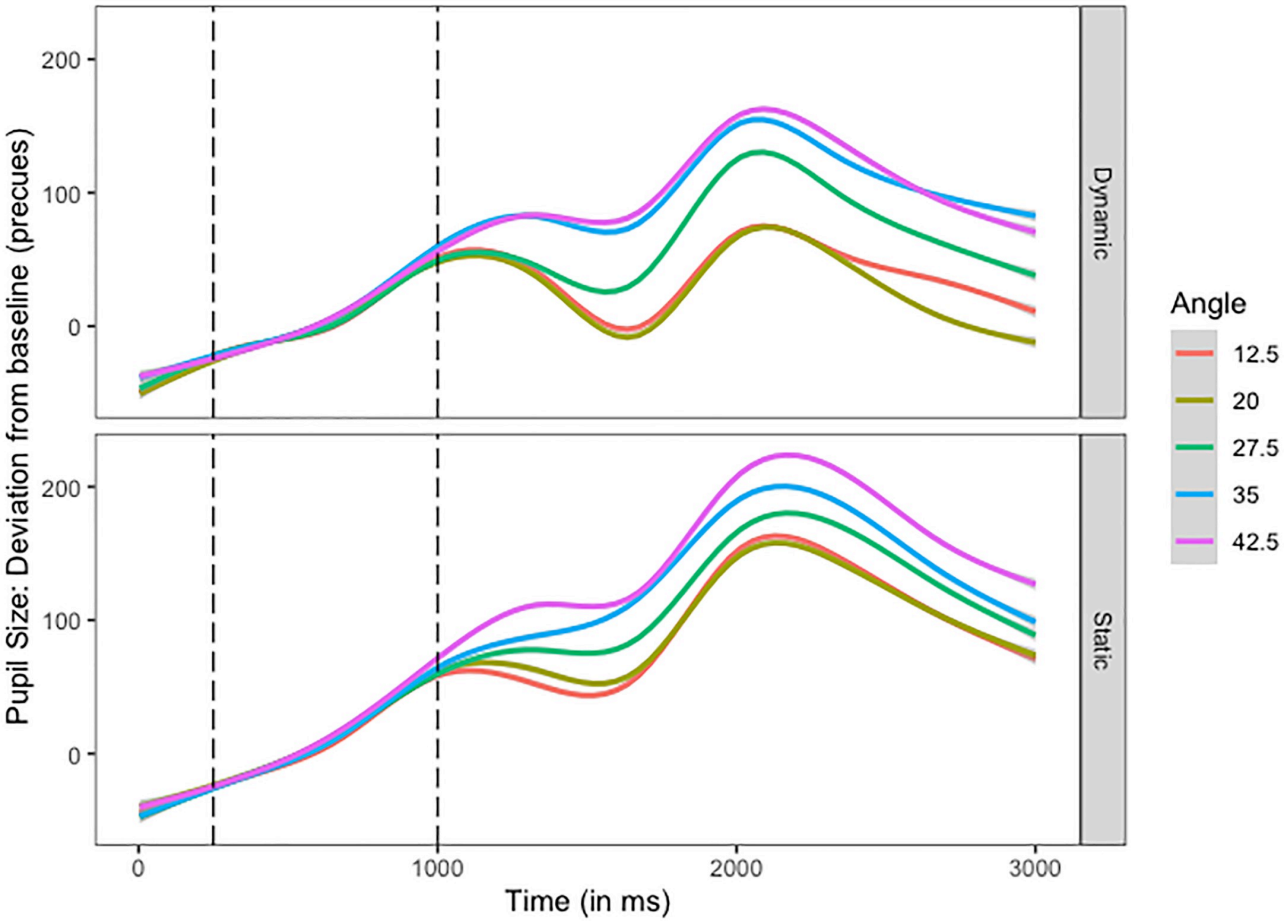
Timeline of the mean pupil size deviations (in arbitrary units) from the baseline per angle, in number of pixels starting at the beginning of a trial (cf. Fig 1). Each angle is represented as a separate line. The dashed vertical lines represent the onsets of the pre-cues and the measurement phase, respectively.

The timeline of the baseline-corrected pupil dilations are plotted in Fig 3. As the plot shows, pupil dilation increased with increasing angle, just like it was observed by Brocher et al. [7] with their static stimuli. In addition, pupil dilation was much stronger in the static than the dynamic mode for all five angles. This was, however, not supported by results of an additional 2 (presentation mode: static, dynamic) x 5 (angle: 12.5°, 20°, 27.5°, 35°, and 42.5°) repeated measures ANOVA on the pupil size data. There was no effect of presentation mode, *F*(1, 19) = 2.203, *p* = .154, or angle, *F*(4, 76) = 0.049, *p* = .154, and also no interaction of both factors, *F*(4, 76) = 2.408, *p* = .057.

Nevertheless, statistical analyses revealed that the model including presentation mode as predictor (AIC = 40206), yielded a better fit to the data than the intercept-only model (AIC = 40214), Χ^2^(1) = 9.68, *p* = .002, meaning that static vs. dynamic stimuli presentation differently affected the size of the pupil. Also, including the angle further improved model fit (AIC = 40163), Χ^2^(4) = 51.93, *p* < .001, (*R*^*2*^ = 0.02, *b*_*0*_ = 247.82, *b*_*presentation mode*_ = -54.165, *b*_*angle*_ = 11.22, *ps* > .09). This suggests that pupil size was additionally affected by the distance between the stimuli and eye fixation. The inclusion of the interaction term did not improve the model fit any further, Χ^2^(4) = 1.17, *p* = .883, (AIC = 40169).

**Fig 4:**
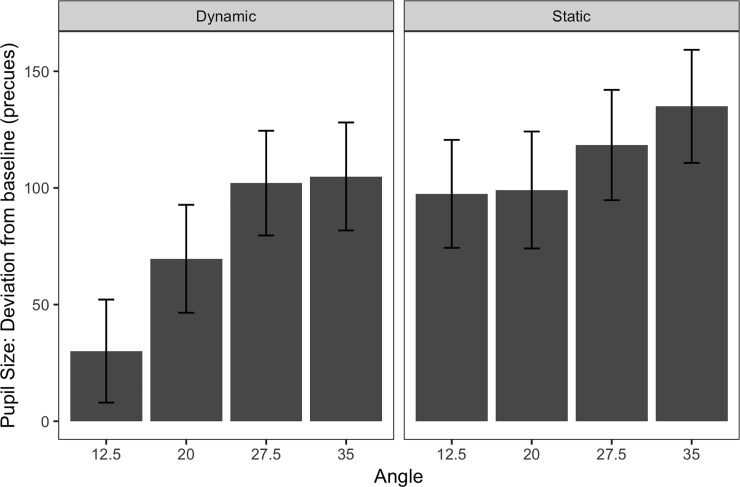
Mean pupil size deviations from the baseline per angle when comparing the starting points of the moving stimuli in the dynamic mode with the respective angle of the non-moving stimuli in the static mode. Error bars provide 95% confidence intervals.

Before we turn to the discussion, we provide two additional pieces of information. First, one might argue that the starting points of the moving stimuli in the dynamic mode were always closer to eye fixation than the starting (and end) points of the static stimuli. This could have led to smaller pupils in the dynamic mode in comparison to the static mode, as inspection might be easier when the first visual contact occurs closer to the fixation point than when it occurs farther away from eye fixation. Although, the timeline in Fig 3 already reveal that the smallest angle in the static mode yielded a mean peak that is comparable to the largest angle in the dynamic mode, Fig 4 shows even more clearly that when we compare the starting points of the dynamic mode with the respective angles of the static mode, pupils still dilated much more strongly for each angle in the static than in the dynamic mode.

Second, because stimuli closer to the central fixation point possibly have stronger effects of luminance on the pupil dilation than stimuli farther away from the fixation point, it is plausible that the correlation of angle and pupil dilation was (also) due to differences in eccentricity (see [7] for a longer discussion). If this were the case, we would expect much larger differences between particularly bright and dark stimuli for the smaller angles compared to the larger angles, as differences in luminance should then decrease as the respective stimuli move away from eye fixation. To test this, we conducted another 2 (number of white objects: zero vs. eight) x 5 (angle: 12.5°, 20°, 27.5°, 35°, and 42.5°) repeated measures ANOVA on the pupil size data of the dynamic mode condition. There was no significant effect of the number of white objects, *F*(1, 19) = 1.8, *p* = .196, and also no interaction effect involving the number of white objects, *F*(4, 76) = 2.118, *p* = .087. That is, there seemed to be no systematic relation between the number of white objects and pupil dilation for the five angles, although we do note that, for the 20° and 27.5° angles, stimuli with eight white objects yielded the weakest dilations.

## Discussion

In this study, we used an attention-window task to investigate if the pupil size can be used as an indication of whether participants attend to stimuli that move vs. do not move in their peripheral vision. Our rationale was that if this was the case and the pupil size was found to be a valid indicator, the attention-window task might be a valuable tool for research on specific properties of the visual attentional system. As we pointed out earlier, the use of the task and the measurement employed in the current study has one great advantage. Both can be adopted in experiments in which the participants can self-determinately choose which object(s) to attend to, rather than being explicitly instructed on which object(s) to attend to if future studies in which participants are less restricted in their allocation of attention confirm the current results. In other words, if attentional shifts to objects in the peripheral vision are measured through response accuracy alone, we typically need to make sure that we know in advance which object(s) the participants will attend to, as response accuracy is the only measure of attentional shifting.

On a more general level, if we assume that the observed differences in the pupil size during the inspection of static and dynamic objects are due to differences in the exerted effort on the task (cf. [7]), the results of the present study might be taken as an indication that tasks involving attentional shifts to dynamic stimuli require less effort than tasks involving shifts to static stimuli (contra [18]). However, again, our experiment was not designed to address this question, but was instead conducted to provide empirical evidence that we can use pupil dilation to track attentional shifts to dynamic vs. static objects. Taken together with the results reported in Brocher et al.’s [7] study, we summarize that pupil dilation can reflect differences in attention to close vs. distant stimuli, stimuli of high vs. low contrast, and, as confirmed by the present results, dynamic vs. static stimuli. Lastly, pupil dilation also seems to indicate whether an observer directs attention to an object or whether she or he merely detects stimuli in their peripheral vision.

Another interesting outcome is that the pupil dilation decreased when dynamic stimuli, instead of static stimuli, were presented, (as if attending to the moving stimuli required less mental effort) although, the accuracy decreased as well. This finding is not in line with previous findings which state that invariably, not only manipulations increased difficulty (as indexed by more frequent errors), but also yielded larger pupil dilations when moving objects were presented [10, 22].

There are some limitations of the present study and considerations for future research that should be addressed. In the current study, the eccentricity of target stimuli was not matched between the two modes: the moving stimuli appeared 7.5 degrees closer to fixation and ended at the same eccentricities as the static stimuli, and were therefore, on average, closer to the fixation point. It can be argued that the difference in eccentricity by itself is insufficient to account for the difference between pupil dilations in static vs. dynamic modes. However, we did not consider that the effect of eccentricity on pupil dilation (and accuracy) does not necessarily need to be a linear function of the eccentricity angle: moving the target closer to the fixation point by 7.5 degrees of visual angle might have a larger effect when it is done in relation to a position at 12.5 degrees than, say, a position at 35 degrees of visual angle. However, as shown in Fig 4, at least for some of the values, the results seem to roughly point to a linear relationship of eccentricity and pupil dilation: The pupil response to moving targets, which started at 20 degrees and terminated at 27.5 degrees, was roughly the same as the response to the static target at 20 degrees. Future studies are needed to exclude that the difference in pupil dilation across static and moving stimuli is not due to an eccentricity error, but rather due to the fact that pupil dilation does discriminate between moving and static stimuli.

In addition, pre-cues in the static condition might have been more valid than pre-cues in the dynamic condition (especially during the first trials of every participant). That is, the circle cues in the static condition appeared in the center of where subsequent target stimuli would appear. That is not the case in the dynamic condition, in which the circle cues appeared at the end position of stimulus movement, thus not where stimuli would initially appear. The former may be associated with less effort than the latter. To effectively rule this out, future should change the way pre-cues are used in the dynamic presentation mode.

Furthermore, future studies should differentiate between the movement direction of the dynamic stimuli so that they do not always move away from the center but also towards the center (i.e., from inside to outside, but also from outside to inside). Also, the target stimuli should not always move from one angle to the next, as was in the current study, but should also move across multiple angles and at different velocities. Another recommendation for future research would be to use some kind of a moving object, like in phase-shifting Gabor (cf. [23]), or a rotating pattern, that does not change position at all, in order to circumvent the moving distances and potential eccentricity biases.

Of course, any experiment that implements pupil size as a measure of attentional shifting needs to include thorough initial calibration and balancing of trials across the conditions under investigation. In particular, when participants are free to pay attention to their chosen object(s) from the available objects onscreen, one needs some initial measure that links the various conditions to the size of the pupil. For example, if in future studies we were to present four objects in peripheral vision at the same time and aim to investigate which object(s) did the participants attend to, we would need a sufficient number of initial trials, in which we control object(s) which the participants attend to (as was done in the current study). Only then are we able to compare the pupil size in free-choice trials with the pupil size in forced-choice trials, and determine which object(s) the participants attended to in the free-choice trials from that comparison. Such a design, we propose, should be the next step in the implementation of pupil size as a measure in investigations on attentional shifts to peripheral vision.
